# Liquid Baits with *Oenococcus oeni* Increase Captures of *Drosophila suzukii*

**DOI:** 10.3390/insects12010066

**Published:** 2021-01-13

**Authors:** Gordana Ðurović, Amani Alawamleh, Silvia Carlin, Giuseppe Maddalena, Raffaele Guzzon, Valerio Mazzoni, Daniel T. Dalton, Vaughn M. Walton, David M. Suckling, Ruth C. Butler, Sergio Angeli, Antonio De Cristofaro, Gianfranco Anfora

**Affiliations:** 1 Research and Innovation Centre, Fondazione Edmund Mach, 38010 San Michele all’Adige, Italy; valerianaof@gmail.com (G.Ð.); silvia.carlin@fmach.it (S.C.); valerio.mazzoni@fmach.it (V.M.); gianfranco.anfora@unitn.it (G.A.); 2 Biobest Group NV, Ilse Velden, 2260 Westerlo, Belgium; amani.alawamleh@biobestgroup.com; 3 Department of Agricultural, Environmental and Food Sciences, University of Molise, 86100 Campobasso, Italy; peppemad@hotmail.com; 4 Technology Transfer Centre, Fondazione Edmund Mach, 38010 San Michele all’Adige, Italy; raffaele.guzzon@fmach.it (R.G.); max.suckling@plantandfood.co.nz (D.M.S.); 5 Department of Horticulture, Oregon State University, 4017 Ag and Life Sciences Bldg., Corvallis, OR 97331, USA; daniel.dalton@oregonstate.edu (D.T.D.); vaughn.walton@oregonstate.edu (V.M.W.); 6 Biosecurity Group, The New Zealand Institute for Plant and Food Research Limited, PB 4704, Christchurch 8140, New Zealand; Ruth.Butler@plantandfood.co.nz; 7 School of Biological Sciences, The University of Auckland, Auckland 1010, New Zealand; 8 Faculty of Science and Technology, Free University of Bozen-Bolzano, Piazza Università 5, 39100 Bozen-Bolzano, Italy; sergio.angeli@unibz.it; 9 Centre Agriculture Food Environment (C3A), University of Trento, 38100 San Michele all’Adige, Italy

**Keywords:** spotted-wing drosophila, invasive species, feeding attractant, lactic acid bacteria, insect trapping, fruit fly lure, volatile organic compounds

## Abstract

**Simple Summary:**

Among the challenges arising from climate change and the transformation of agroecosystems is that agricultural production is heavily affected by invasive insect species. Invasive insects can establish in new areas where their development can progress due to a suitable climate and lack of natural enemies. Farmers have few options to mitigate those insects’ attacks. Current control tactics using pesticides must be replaced with more sustainable methods to counter invasive insect species. We approached the control of the invasive spotted-wing drosophila *Drosophila suzukii*, using a baiting system that manipulates insect behavior without use of toxic or non-sustainable chemicals. The results of our work are utilized for the monitoring and mass trapping of this devastating invasive species. In our innovative smart-design trap system, we use odors that attract flies and decrease damage in open field scenarios. Our trapping system can efficiently detect the first spring arrival of *D. suzukii* in agricultural fields and as a such, represents a good early monitoring tool. We conducted four years of laboratory and open-field trials in different berry crops. As a source of odor attraction, we used a mixture of wine, apple cider vinegar, and different commercially available strains of lactic acid bacteria.

**Abstract:**

The spotted-wing drosophila (SWD), *Drosophila suzukii* Matsumura (Diptera: Drosophilidae), native to Eastern Asia, is an invasive alien species in Europe and the Americas, where it is a severe pest of horticultural crops, including soft fruits and wine grapes. The conventional approach to controlling infestations of SWD involves the use of insecticides, but the frequency of application for population management is undesirable. Consequently, alternative strategies are urgently needed. Effective and improved trapping is important as an early risk detection tool. This study aimed to improve Droskidrink^®^ (DD), a commercially available attractant for SWD. We focused on the chemical and behavioral effects of adding the bacterium *Oenococcus oeni* (Garvie) to DD and used a new trap design to enhance the effects of attractive lures. We demonstrate that microbial volatile compounds produced by *O. oeni* are responsible for the increase in the attractiveness of the bait and could be later utilized for the development of a better trapping system. Our results showed that the attractiveness of DD was increased up to two-fold by the addition of commercially available *O. oeni* when combined with an innovative trap design. The new trap-bait combination increased the number of male and especially female catches at low population densities.

## 1. Introduction

*Drosophila suzukii* Matsumura (Diptera: Drosophilidae), the spotted-wing drosophila (SWD), is an invasive insect species that first appeared in Europe and North America in 2008 [1]. Originally from south-east Asia, *D. suzukii* was quickly established in this new range that had a suitable climate for rapid development and contained few natural enemies [2]. Evolutionary flexibility and a fast life cycle have allowed *D. suzukii* a successful fast invasion and spread. Females have a sclerotized, serrated ovipositor, allowing them to lay eggs under the skin of host fruits, while males have a dark spot on the outer margin of each wing [3,4]. These sexually dimorphic characteristics allow for the differentiation of *D. suzukii* from other species of *Drosophila* [3,5]. Developing larvae feed on the pulp of the fruit, resulting in economic damage. Oviposition activity further opens a route of infection for pathogenetic microorganisms [1]. The fruit becomes increasingly susceptible as harvest time approaches; thus, few chemical interventions are left as an option because of the likely presence of pesticide residues. A broad range of soft-skinned host fruits is available for attack by *D. suzukii* [6,7,8]. Furthermore, *D. suzukii* is a mobile pest that is able to shift environments from high woody bushes in winter to crops in warmer months during times of host fruit availability [9,10].

Volatile organic compounds (VOCs) are abundantly produced by microorganisms, plants, insects, and all organisms that are part of terrestrial or marine ecosystems [11]. Ecological roles of VOCs are broad, and yet their functions in chemical communication between organisms largely remain to be investigated [12]. VOCs mediate host preference [13], mate location and ovipositional site choices of *D. suzukii* [14,15,16]. The attractive VOCs are products of microorganisms that cohabitate on surfaces of host plants within the insect habitat [17,18,19]. In particular, microorganisms such as bacteria, fungi, and yeast produce distinct and abundant microbial volatile organic compound (MVOC). MVOCs produce odors that influence complex behavioral interactions between insects and their habitats, yet little is known about how organisms exploit such compounds as behavioral cues [20]. Microbes make significant contributions to plant volatile blends. While the role of microbial volatiles in plant-insect interaction is to some extent summarized, an increasing field of research on MVOCs and their full potential in practical application is yet to come [20]. Microorganisms present on the surfaces of their host plants produce MVOCs that are important for the attraction of insects also affect insects from other trophic levels resulting in multi-trophic interactions [21,22].

Such volatile compounds can be exploited for the development of SWD control methods [23,24]. SWD are attracted to the MVOCs from fermenting substrates for feeding purposes [25,26]. At present, the most attractive traps for *D. suzukii* are those baited with vinegar and wine. Such traps serve as one of the first means for early detection and mass trapping [27,28,29,30]. Monitoring is the first step in an integrated pest management (IPM) program, allowing for determining where populations are present in the field, and when to enact control measures [29,31]. Currently, an accepted and commonly used monitoring method for *D. suzukii* involves the use of traps constructed from plastic cups with holes at the top for fly access (i.e., Droso Trap^®^, Biobest, Westerlo, Belgium; Victor^®^ Poison Free^®^ yellow jacket and flying insect trap, Great Lakes IPM Inc., Vestaburg, MI, USA). Inside the cup there may be a yellow sticky card and/or a mixture of apple cider vinegar (ACV), wine, vinegar and other fermenting liquids, and a surfactant [26,27,32,33,34]. However, these traps can often only detect insect activity after that the seasonal insect population has already established. Consequently, the suppression of the pest population is a serious challenge [35]. Another drawback of these traps is that they are not species-specific and hence there is a need to create a more sensitive trap. Understanding the biology and ecology of *D. suzukii* is therefore fundamental to setting up and optimizing control techniques based on the interference of insect behavior [36].

Some information is known about bacterial volatiles and their influence on the attraction of *D. suzukii* [19,21]. With regard to symbiotic bacteria, the species with the highest frequencies in communities associated with *D. suzukii* are *Tatumella* spp. (Enterobacteriaceae), *Gluconobacter* spp., and *Acetobacter* spp. (Acetobacteraceae) [37]. Certain bacterial species were shown to produce volatile metabolites capable of attracting *D. suzukii* in olfactometer assays [19]. However, to date, there is little evidence that lactic acid bacteria (LAB) involved in the fermentation of wine or vinegar increase attractivity. 

There are several studies on sugar fermentation by both yeasts and LAB and the effects they have on wine. Yeasts play a significant role in attraction of *D. suzukii* [38,39]. Currently, the most important synthetic attractants for *D. suzukii* contain synthetic VOCs identified in the headspace of wine, vinegar, apple cider, and rice vinegar; such compounds are produced as a result of yeast metabolic fermentation on or within host fruits [28,30,40,41]. In particular, [42,43] confirm the importance of the LAB species *Oenococcus oeni* (Garvie) in wine production. Cultures of *O. oeni* are found in certain wines [42,43]. Large variability, in terms of resistance to environmental limiting factors and biosynthetic capacity, has also been reported between separate strains of *O. oeni* [44]. Therefore, we decided to conduct detailed investigative studies on MVOCs produced from fermentation by LAB.

Strains of LAB were selected based on their commercial use in the fermentation of wine and vinegar, and on their resistance to stressful environmental conditions present in the liquid baits. We focused our attention on the interactions between *D. suzukii* and *O. oeni*, with the aim of developing a new trap capable of early fly capture.

The main objective of this study was to improve the existing trapping and monitoring system of *D. suzukii* in an open field setting. Previous studies on the attraction of *D. suzukii* have focused on volatiles from the yeast fermentation of host fruits. We focused on VOCs produced as a result of bacterial fermentation. Bacterial strains were chosen under laboratory conditions and then further tested in preliminary field trials to determine the best strain of lactic acid bacteria (data available on request). In this study, field trials conducted in Oregon, USA assessed the performance of different trap models baited with the selected commercial biotypes of *O. oeni*. Additionally, we aimed to identify the chemical cues from bait mixtures that elicited a strong attraction response in *D. suzukii*. To test these mixtures, we used a combined approach of gas-chromatography and electrophysiology. Therefore, quantitative variations induced in the Droskidrink (DD) (combination of 74.5% apple cider vinegar, 25% red wine, 5 g L^−1^ sugar cane) by the addition of bacterial stains both in the case of an increase and decrease of the initial amount of each volatile compound, were carefully considered. We found an unexpected set of chemical compounds produced as products of bacterial fermentation. Our field studies showed that the attractiveness of DD was increased up to two-fold by the addition of commercially available strains of *O. oeni*, when combined with an innovative trap design.

## 2. Materials and Methods 

### 2.1. Bacterial Strains and Composition of Droskidrink for D. suzukii Trapping

We conducted three field experiments using DD mixture with following composition: 20 g of sugar dissolved in 750 mL apple cider vinegar (Sysco Corporation, Houston, TX, USA), 250 mL Merlot red wine (Peter Vella, Modesto, CA, USA), and a drop of soap and either Enoferm Alpha or Enoferm Beta of *O. oeni* (Lallemand Inc., Montreal, QC, Canada). The mixtures were left to ferment for one week prior to use in the field trial.

### 2.2. Trapping Experiments with Fermentation in an Open Field

After the preliminary field experiments were conducted, three further experiments were carried out in a blueberry field site in Salem, OR, USA (44°54′34″ N; 123°06′51″ W). The objectives were to find an improved trap design and identify an effective lure.

#### 2.2.1. Trial 1: Comparison of New Traps

The aim was to search for a new trap model that could capture higher numbers of SWD. In this trial, the fermentation process was carried out in the field using two commercial strains of *O. oeni* that showed the best growth performances in preliminary open field trials. In this trial, we wanted to determine which combination of *O. oeni* strain and trap performed best. In order to create an optimum environment for the colonization and reproduction of the bacteria, the pH of the liquid lure was raised to 3.8 using potassium hydroxide (KOH) (monohydrate granular AR [ARS]). Eight treatments were tested in Trial 1 (Table 1). 

Treatment A represented the standard DD bait. Treatment B comprised the standard bait DD with the pH increased to 3.8. Treatment C was the commercially developed Cha-Landolt bait (Pherocon SWD dispenser, Trécé Inc., Adair, OK, USA). Treatments D and E were the same as B but additionally inoculated with 0.5 g L^−1^ of Enoferm Alpha and Enoferm Beta, respectively. Treatment F was the same as Treatment E, but with the addition of 1.0 g L^−1^ of citric acid (pellets AR [ARS]). Adding citric acid to the mixture results in malolactic fermentation that leads to the increased production of acetoin, diacetyl (also called 2,3-butanedione) and 2,3-butanediol [43]. These compounds may increase SWD attraction to the trap. Treatments G and H had the same composition as Treatment E, but the trap designs were different. 

For Treatments A–F, the traps comprised a red plastic cup (532.3 mL) with a white lid (Picture S1c). Polystyrene cups were placed inside each of these to provide insulation. The temperatures of the liquid baits were monitored with portable data loggers (HOBO pendant loggers, Onset Computer Corporation, Bourne, MA, USA). To provide access for SWD, six holes (0.5 cm diameter) were punched into each cup. Cups were filled with approximately 200 mL solution of the appropriate treatment. 

For Treatments G and H, corrugated plastic delta traps (Suterra LLC, Bend, OR, USA) containing a white sticky card were used. The delta traps were modified by creating a hole at the bottom center of the traps, into which the head of a water bottle (Camelbak, Petaluma, CA, USA) was inserted (Picture S1 DIB). This bottle contained a pressure valve that allowed for the release of volatile compounds during fermentation. For Treatment G, insulated bottles were used; for Treatment H, the bottles were not insulated (Picture S1).

The treatments were replicated four times and laid out in a randomized block design, with one replicate per row, and a distance of about 10 m between traps. The experiment was conducted in the center of the blueberry field in Salem, Oregon, USA, avoiding the perimeters, to ensure a more homogenous olfactory environment. The experiment was conducted over four weeks (from 28th August to 25th September 2015) and checked on a weekly basis. Precipitation during the 28-day period totaled 24 mm. The traps were filtered, and the contents returned to the lab in 70% EtOH. Sticky cards (Treatments G and H) were covered with plastic film and transported to the laboratory. Male and female SWD were identified and counted using a dissecting microscope.

The baits for Treatments A and B were replaced weekly. The baits for Treatments C to H were left in the field for the duration of the experiment. The temperatures of the bait in these treatments were monitored with portable data loggers to check if the conditions allowed fermentation (mean = 19.1 °C, minimum = −0.4 °C, maximum = 56.9 °C).

#### 2.2.2. Trial 2: Fermentation in the Open Field and a Different Trap Design

In the second stage of the fieldwork (Trial 2), the liquid bait was replaced on a weekly basis for all treatments. In addition, the amount of bacterial inoculum used for the preparation of the treatments was reduced from 0.5 g to 0.2 g L^−1^. The fermentation of DD by *O. oeni* was conducted in the laboratory for one week prior to the field trials under a controlled temperature of 22 ± 2 °C.

Eight treatments (see Table 1) were tested. The same trap designs were used for treatments A–F as in Trial 1; traps for treatments G and H were modified to allow the insertion of 30-mL cups (Dart Container Corporation, Mason, MI, USA) in the center and the water bottles were removed. For Treatment G, about 20 mL of DD was added to each trap. The cups were closed with specially prepared covers that excluded entry of insects but allowed volatile compounds to escape (Picture S1 DC a). For Treatment H, approximately 15 mL of DD per trap was used, and two cotton balls were inserted inside the cups, which were left without lids (Picture S1 DC b).

Trial 2 was carried out in the same blueberry field as Trial 1. Four replicates of each treatment were used and laid out in a randomized block design similar to Trial 1. The trial was conducted over four weeks (from 25 September to 23 October), traps were checked on a weekly basis, and captured species were identified in the laboratory. Precipitation over this trial period totaled 21 mm.

#### 2.2.3. Trial 3: Evaluation of the New Trap Design

Trial 3 was the last phase of the fieldwork and involved the assessment and development of a different trap as well as the improvement of traps used in previous trials. The aim of this trial was to assess whether this new model of the trap could further enhance the results achieved by the mixtures of DD inoculated with bacteria.

This trial used six treatments (see Table 1). Treatments A, B, and C were as described previously, using the standard red cup trap with a white lid. Treatments D, E, and F used the same lures as for treatments D, E and F in Trial 2, and, lures were used, with the plastic delta trap with a 30-mL cup inside (Picture S1 DC b). This cup was equipped with two cotton balls and 15 mL of the test mixture. This trial took place over 5-weeks following the dry summer period, 30 October to 4 December 2015, (total precipitation was 128 mm of rain). During this time, temperatures were cooler than in previous experiments (mean = 8.1 °C with minimum −10.3 °C and maximum 32.6 °C) and, consequently, the population of SWD was relatively low. Once again, traps were checked weekly, and insects were counted. Three replicates of the treatments were used and laid out in a randomized block design, similar to the previous trials.

### 2.3. Statistical Analysis

For all trials described in Section 2.2.1, Section 2.2.2, and Section 2.2.3, the number of SWD caught on a trap across all assessments was calculated for females, males and total (male + female). For each category (males, females and total), the numbers of SWD were analyzed with a Poisson generalized linear model (GLM) [45] with a log link. Proportions of females were analyzed with a binomial GLM with a logit link. For all analyses, there was substantial over-dispersion; thus, the dispersion was estimated (i.e., quasi-Poisson and quasi-binomial GLM were used). An overall assessment for treatment differences was carried out using an F-test as part of the analysis of deviance within the analysis. Mean counts and percentages were obtained on the log/logit scale, along with associated 95% confidence limits; these values were back transformed. The means and confidence limits were then divided by the number of days that had elapsed since trial set-up to give mean catches per trap per day. All analyses were carried out with Genstat [46].

### 2.4. Headspace Collection and Chemical Characterization of DD Inoculated with Different Strains by GC-MS 

To characterize most volatile compounds of DD alone and DD with the addition of *O. oeni* reference strain MRI1000, Enoferm alpha, Enoferm beta, T1, S18, P1 was performed using dynamic DHA (direct headspace analysis) collection connected to gas chromatograph (GC). Samples of DD were inoculated with 1% *v*/*v* bacterial cultures and incubated at 25 °C for one week. Prior to the incubation, 5 mL of DD were inserted into hermetically sealed glass vials (20 mL), and the volatile compounds were introduced to the analyzer using a multifunctional autosampler (Multi-Purpose Sampler MPS, Germany). Each vial was automatically introduced into an incubator at 38 °C for 20 min, then 1 mL of air was removed from the headspace using a gas syringe preheated to 80 °C and subsequently injected into the column of the GC. The GC-MS analysis was performed using a 7890 A Gas Chromatograph (Agilent Technologies, Santa Clara, CA, USA) equipped with an HP-5ms column (length = 30 m, internal diameter = 0.25 mm, with a 0.25 μm film thickness) (Agilent Technologies), coupled with a 5975 inert XL mass selective detector (Agilent Technologies). The method was set to see ions in the range of 34 and 300 a.m.u. Sample introduction was performed by a multifunctional autosampler, and samples were prepared by the robot (MultiPurpose Sampler MPS for GC and GC/MS, Gerstel, Mülheim an der Ruhr, Germany). Helium was used as the carrier gas (flow rate of 1.2 mL min^−1^); the thermal cycle provided was 5 min at 30 °C, a temperature ramp of 3.5 °C min^−1^ to 240 °C, and 2 min at 240 °C. The total run time was 44.87 min, in splitless mode, total flow 54.2 mL min^−1^, purge flow to split vent 50 mL min^−1^ at 1 min temperature program 240 °C for 0 min. The volume injected was 2.0 µL, acquisition mode was scan, scan parameters 35 to 400 a.m.u. Solvent delay of 4 min. The extracted ions are used for quantification of each single compound. Data acquisition and analysis were done by MassHunter Software (Agilent Technologies, Santa Clara, CA, USA).

### 2.5. Droskidrink Preparation with Oenococcus oeni Strain Pn4 (Alpha) and Strain 31 (Beta) for VOC Collection 

Selected strains of *O. oeni* were added to DD at a rate of 1 g per 400 mL liquid bait and homogenized by stirring. The pH of the mixture is normally ~2.5; for the trials, KCl was added until pH reached 4.5. The mixture was prepared in lidded cylindrical polyethylene boxes 500 mL in volume. The mixture was left under aerobic conditions with a photoperiod of 16:8 light:dark (1000 lux during the light period) at 25 ± 2 °C and 60 ± 5% relative humidity for 7, 14, or 21 days, after which the elution of volatiles was performed.

#### Volatile Collection

Aeration and adsorption were performed as described in [47] as direct head space (DHS). Briefly, the odor source, a mixture of DD, and a mixture of DD + LAB were placed within an aeration chamber. Air entering the vessel was pulled through a filter of activated charcoal and allochroic silica gel and was then pumped out of the vessel for 48 h at 0.4 L min^−1^ through an absorbent tube. Volatiles were collected using 8 cm long absorbent glass tubes (5 mm internal diam.), packed with 500 mg Porapak Q polymer (50/100 mesh, Waters Corporation, Dublin, Ireland) between salinized glass wool plugs. Volatiles were eluted from the Porapak Q trap with 1 mL dichloromethane. Three replications of the volatile collections from mixtures of 7-, 14-, and 21-day old fermentations were carried out for 48 h, 10 V in a climatic chamber under a 16:8 h light:dark photoperiod (1000 lux during the light period) at 25 ± 2 °C and 60 ± 5% relative humidity. Nine volatile collections and three replicates of each sample were collected. The collected volatiles were further used in gas chromatography (GC) with use of mass spectrometer detector (GC–MS), GC-EAD-FID analyses and behavioral experiments, and the addition of an internal standard that was subjected to quantitative GC–FID analysis. Volatile samples were stored in 2 mL vials in a cryogenic freezer (−80 °C) until used in chemical analysis and electrophysiological experiments. 

### 2.6. Chemical Characterization of Volatiles from Droskidrink and Oenococcus oeni Strains 

#### 2.6.1. GC-MS Analysis 

GC analyses were carried out with a Trace GC Ultra gas chromatograph coupled with a TSQ Quantum XLS Tandem mass spectrometer (Thermo Electron Corporation, Waltham, MA, USA) and equipped with a PAL Combi-xt autosampler (CTC Analytics AG, Zwingen, Switzerland). The separation module consisted of a ZB Wax PEG capillary column (30 m × 0.25 mm inner diameter × 0.25 µm film thickness; Phenomenex, Italy) programmed to increase from 60 °C (held for 3 min) at 8 °C min^−1^ to 220 °C (held for 10 min) and, finally, to 250 °C at 10 °C min^−1^ for 5 min. Helium was used as the carrier gas at a flowrate of 1.2 mL min^−1^. The temperature of the transfer line was 250 °C. The electron impact energy was 70 eV, and the filament current was 50 μA. The mass range was m/z 40–350 a.m.u. The source temperature was set to 200 °C, the accelerating voltage to 4.6 kV and the filament current in the source set at 1 mA, with little modification as in [9].

#### 2.6.2. Electrophysiological Studies and Chemical Identification of Compounds that Elicited a Response in SWD

The culture of *D. suzukii* used in the laboratory experiments was established from populations collected from orchards located in the province of Trento, Italy. Colonies were reared on a standard Drosophila semi-artificial cornmeal diet, at the temperature of 23–25 °C, relative humidity of 65 ± 5% and with a photoperiod of 16:8 h light:dark [48].

Samples obtained from DHS adsorption eluted in dichloromethane were used for gas chromatography electroantennographic detection (GC-EAD). The procedure involved isolating an antenna (together with head and prothorax), mounting it between two electrodes, applying a current of odorous stimulus from GC-FID (flame ionization detector) and measuring the variation in electrical potential through an oscilloscope. We used a similar procedure to that described in [49].

The GC was equipped with the non-polar HP-5MS column (Agilent Technologies, USA), as described in direct headspace analysis. The samples (2 µL) were injected in spitless mode as described in DHA. The flame ionization detector was set up at 350 °C, with hydrogen flow of 50 mL min^−1^, airflow of 500 mL min^−1^, and helium flow of 20 mL min^−1^. Signal source of FID was 20 Hz min^−1^. Antennal signals were captured using a high-impedance AC/DC pre-amplifier (10×), sent to an IDAC-2 box, and stored on a PC hard disk using EAD2014 software. IDAC 2 was set up at maximum recording duration of 51 min, voltage range 312.5 mV, channel 1 (EAD): low cutoff 0.05 Hz, offset 0, Ext. amp: 23, digital 1 (trigger) initiates recording on, invert on. Channel 2 (FID): low cutoff: DC, offset 0, ext. amp. 10, record signal data on. GC-EAG2014 Sintec software was used for recording and analyzing the data. Mated females aged 7–9 days old were used throughout all experiments. The antennal signals were banded pass filtered between 3 kHz and 0.1 Hz whereas the FID signal was not conditioned; both signals were fed onto separate channels in the IDAC-2, and the digitized signal was fed onto the PC. At least ten flies were tested for each extract odor with up to ten recordings of the same odor per fly. Recordings were performed in the afternoons and typically extended into the evening. The absolute amplitude of the responses was measured in μV from the onset of depolarization (baseline) to the maxima of the deflection [50]. A compound was considered electrophysiologically active when it elicited antennal responses at least three times greater than background noise. Peaks were matched between GC-EAD and GC-MS by the retention index.

### 2.7. Data Analysis from GC-MS and GC-EAD 

First tentative identifications of compounds from entrainment samples focused on peaks that gave a response in GC-EAD were carried out using mass spectrometry (MS) as a detector by the use of a Kovats Retention Index (RI) [51]. To enable the calculation of RI a series of known internal reference standards (alkanes, C7-C25 n-hydrocarbons at 1 μL of 100 ng μL^−1^ diluted in distilled hexane purchased from Sigma Aldrich) were run on the GC columns prior to the analysis of entrainment samples in the same conditions. The Kovats index was calculated and mass spectra were compared with libraries offered by XCalibur 1.2. The calculated Kovats index was compared with those [9,14,49,52] described the electrophysiologically active compounds for SWD and other insect species. If the identification match gave us more than 95% security the compound was tentatively identified.

All components collected from DHS extracts and analysis on the GC-HD-MS were initially tentatively identified after GC-MS analysis, through the comparison of their mass spectra with those present in mass spectral databases. Each peak of interest detected in the gas chromatogram was analyzed and identified through its mass spectra [52]. Because many compounds present in the analyzed samples were concentrated in the first minutes of the x-axis of the chromatograms and were often overlapping, identifications were made on the non-polar column using the technique of single ion extraction, in order to allow a more precise and reliable identification. For quantification, one single ion per compound was “extracted” from the chromatogram and its area quantified for all the samples. The quantity of the detected components was expressed as a percentage to allow easy comparison of differences in VOC emission between DD and DD + LAB.

We performed GC-EAD- to see which compounds elicited an antenna response in *D. suzukii*. Those that were identified as electrophysiologically active compounds were marked, and their linear retention index was calculated. Retention indices from GC-FID and GC-MS were compared, and if they coincided, the compounds were chemically confirmed. Identification of compounds tentatively identified using GC/GC-EAD and GC-MS was confirmed or rejected using co-injection with a laboratory standard on a polar column. The amount of synthetic standard added to the sample was aimed at doubling the area of the peak for that particular substance, without increasing its width. This was achieved by adding the appropriate concentration of a solution of the laboratory standard to the entrainment sample injected into the GC. 

## 3. Results

### 3.1. Trapping Experiments with Fermentation in an Open Field 

#### 3.1.1. Trial 1: Comparison of New Traps

**Figure 1:**
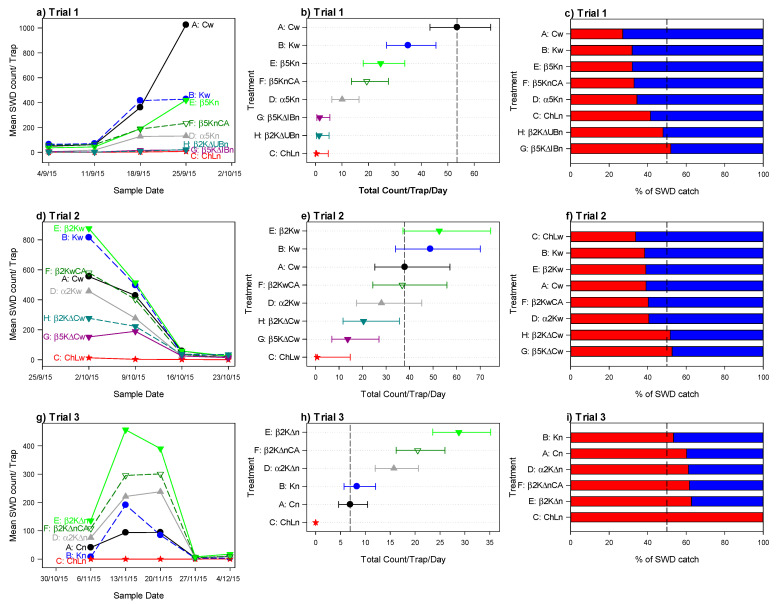
For each treatment in trials 1–3: Mean *Drosophila suzukii* (SWD) total catches per trap at each assessment (**a**,**d**,**g**); dot plots of mean total catch/trap/day with 95% confidence limits (as error bars), sorted by the means, with dotted vertical line at the mean for Treatment A (control) (**b**,**e**,**h**); and percentage of the total catch that was female (blue) or male (red) (**c**,**f**,**i**). Note that for h, the upper confidence limit for a mean of 0 is not shown as it is difficult to obtain. For a description of treatments, see Table 1: Traps are: C = cup with a lid; Δ= delta trap; IB = insulated bottle; UB = uninsulated bottle. Delta traps without a bottle had a cup without a lid and supplied with two cotton balls. Liquid components are: K = KOH added; α= Alpha; β = Beta; 2 = rate 0.2; 5 = rate 0.5; CA = citric acid added. n = no liquid replacement; w = liquid replaced weekly.

In this trial, we assessed the effectiveness of select strains of *O. oeni* in combination with different trap designs. Overall, the total numbers of *D. suzukii* increased from the first to the final assessments (Figure 1). The numbers of males, females, total *D. suzukii*, and the percentage of captured females all varied between treatments (*p* < 0.001, F_7,24_ = 37.8, 33.0, 37.3, 8.3, respectively; Figure 1a–c, Table S1). 

Differences in the numbers of males and females caught were similar across all treatments (Figure S1). The lowest total *D. suzukii* capture rate (six per trap) was for Treatment C, which used the Cha-Landolt bait. The mixtures inoculated with LAB (Treatments E, D, F) were significantly less attractive than standard DD (Treatment A) and DD with pH adjusted to 3.8 (Treatment B). The mixtures inoculated with strains of *O. oeni* were left in the field throughout the 4-week period of the trial, whereas the baits in the control treatments A, B and C were refreshed weekly. This helped to assess the ability of bacteria to perform malolactic fermentation directly under field conditions. In an attempt to create optimal temperatures for malolactic fermentation (20 °C), polystyrene cups were used to buffer against temperature changes. Although the inoculated treatments did capture flies, their poor performance relative to the non-inoculated treatments A and B may be linked to the extreme temperatures reached by the liquids during the night and during the hottest hours of the day. The temperatures of the mixtures fluctuated by as much as 50 °C over the course of the 4-week trial, reaching a minimum temperature of −0.4 °C and a maximum temperature of 56.9 °C. These temperatures are far outside the range required by bacteria to carry out malolactic fermentation.

#### 3.1.2. Trial 2: Fermentation in the Open Fields and Different Trap Design 

In the second trial, all attractants were replaced on a weekly basis. As in Trial 1, numbers of females, males, total *D. suzukii* and the percentage of females varied significantly between the treatments (*p* < 0.001, F_7,24_ = 9.4, 8.6, 9.2, 7.0 for the four variables, respectively, Figure 1d–f). However, the numbers caught per trap per day were generally higher in Trial 2 than in Trial 1 (29.9 individuals trap^−1^ day^−1^ c.f. 18.2 for Trial 1). As in Trial 1, the lowest catch (19 individuals trap^−1^) was for Treatment C, which used the Cha-Landolt bait. However, in Trial 2, total catches decreased from the first to the last week (Figure 1d) (Figure S1d–f).

In Trial 2, the mixtures inoculated with bacteria, particularly Treatment E, had better capture performances than in Trial 1. Treatment E had the highest catch of all treatments, both overall and weekly, with the exception of the last week during which all treatments had low trap captures (Figure 1d). The data therefore suggest that microbial activity, and particularly the activity of LAB, increases the attractiveness of the base DD mix. 

Compared to Trial 1, the total catches in Treatments G and H were higher, although still well below those in the control Treatments A and B. The effective capture that characterizes this model of trap bodes well for its use in future monitoring efforts. 

#### 3.1.3. Trial 3: Evaluation of the New Trap Design

The aim of Trial 3 was to assess whether a novel trap design could further enhance the results achieved by the mixtures of DD inoculated with LAB. Overall, total catches increased from week 1 to weeks 2 and 3, and then dropped to almost zero for the final 2 weeks of the trial (Figure 1g). Numbers of males, females, total SWD, and the percentage of females caught all varied substantially between the treatments (*p* < 0.001, F_5,12_ = 34.4, 43.7, 40.6, F_5,10_ = 5.2; Figure 1g–i;) for the four variables, respectively. Two traps had no catches (total SWD = 0). Differences in the number of trapped males and females was similar between treatments. The lowest catch (0.3 trap^−1^) was for Treatment C.

For Trial 3, the modified delta trap (as used in treatment H of Trial 2) was evaluated using different modifications of the DD mixture. When compared to the controls (treatments A, B, and C), all treatments caught on average at least twice as many insects. This differs from results in Trial 1 where the control (treatment A) caught the largest numbers, and in Trial 2 where only treatments B and E caught more insects than the control. Thus, the results of Trial 3 provide further support that the trap design has been improved. Mixtures inoculated with LAB provided excellent results throughout the five weeks of the trial. In Trial 3, more than 50% of the catch were females for all treatments. This is in contrast to the first two trials, where more males than females were caught for almost all treatments. Additionally, in this new trapping system, the time required for identification and counting of insects was one third of the time needed for the previous standard traps. The best results were achieved using treatment E, but all the treatments inoculated with LAB outperformed the control treatments that used the standard cup trap (Figure 1h). This same pattern of increased capture with the new trap design was present each week where catches were non-negligible (Figure 1g and Figure S1g–i).

### 3.2. Headspace Characterisation of DD Inoculated with Different O. oeni Strains by GC-MS 

We selected the six strains of *O. oeni* (MRI1000, alpha, beta, T1, S18, P1) for the analysis of volatile organic compound (VOC) release from the mixture of DD and *O. oeni* strains, which we compared with a standard unfermented DD sample (Table 2). The aim was to detect the most volatile compounds in the mixtures that in standard GC-MS normally were hidden behind solvent peaks. The MRI1000 strain was analyzed as a reference strain of *O. oeni*. Thirteen compounds were detected (Table 2); however, no qualitative differences were found among the baits, and the same compounds were detected in most samples. Interestingly, the relative quantity of compounds varied between samples. The T1 strain was characterized by a high concentration of acetic acid and ethanol. The beta and S18 strains showed a considerable reduction of acetic acid emission (−45% of DD release), while ethanol increased by about 30%. T1 and P1 strains produce a very high quantity of acetic acid, 874% and 669% more, respectively, than the DD alone. They also showed higher ethanol content than the other samples. For the alcohol 3-methyl-1-butanol, all baits showed a content equal to or higher than 100%, except the S18 sample (85%), compared to DD alone. The bacterial strains T1 and P1 produced only 79% and 44% less acetoin, respectively, compared to DD alone. For the compound acetidin (ethyl acetate), the content of all bacterial baits was very low compared to that of DD. The beta strain has a content of 7% and alpha 6%, while strains T1, S18 and P1 are around 4%, relative to DD alone.

### 3.3. Chemical Characterisation of Volatiles from Droskidrink and O. oeni Strains 

Gas Chromatography (GC) on the polar column: GC analysis of the entrainment samples of DD and LAB revealed a complex mixture of volatile compounds that change over time and with use of different bacterial strains (Figure S2). The experiments also revealed complex changes in the quality of the VOCs released from tested mixture during a three-week period (Figure S3a–c, Figure S4). We tentatively identified about 80 peaks from GS-MS data sets using the non-target approach with data analysis software Xcalibur 1.2 (data available upon request). GC co-injection with synthetic chemical standards confirmed the identity of target compounds present in the entrainment samples in areas of 24 peaks. The remaining 7 peaks that elicited a response in electrophysiological studies were only tentatively identified because of the absence of laboratory synthetic standards or the presence of different isomers. GC-EAD-FID identified 32 active EAD peaks in the different samples of DD and samples with LAB (Table 3). 

It is evident that bacteria increase the quantity of some VOCs in the mixture (Figure S2a–c). The complex, volatile blend inside mixtures changed not only in the presence of LAB, but also with the aging of the mixture. Most compounds of interest were not detectable 20 days following addition of bacterial strains. In general, the amount of total alcohols in DD with LAB increased in the second week, then decreased by the third week. Acid compounds decreased toward the second week, and then later increased. Addition of *O. oeni* strain 31 to DD increased the total amount of acid compounds together with alcohols, while esters and ketones were reduced (Figure S5a–c). *Oenococcus oeni* strains 31 and PN4 showed a considerable reduction of acetic acid and acetoin in the second week (Figure S3b,c). The emission rate of 3-methyl-1-butanol, reported as a “non-target” attractive volatile for a wide range of moths [53], was higher than in the standard DD in the case of *O. oeni* strains 31 and PN4. 

### 3.4. Results from Electrophysiological Experiments

GC-EAD and compound identification by GC-MS and GC-co-injection: GC-EAD analysis of responses was constant and significantly higher than background noise in the 32 EAG peaks in the entrainment samples of DD and DD + *O. oeni* strains 31 and PN4 (Table 3). Confirmation of the identity of the electrophysiologically active compound with RT 12.42 on the non-polar column could not be made, therefore the compound is not presented in Table 3. This compound according to mass spectra is a monoterpene, but it did not match any library search. Also, the compound on the non-polar column that elicited a response with RT 7.006 could not be identified, although a NIST11 (National Institute of Standards and Technology, Gaithersburg, MD, USA, 2011), Wiley7n, and W9N08library search with 90% of match offers identification as 3-Hexenoic acid, ethyl ester. The laboratory standard of this compound is however not available. 

## 4. Discussion

Our results show the basis for improved attraction of spotted wing Drosophila using lactic acid bacteria (LAB) fermentation acting on a complex mixture of apple cider vinegar, red wine, and sugar cane. The addition of fermentation by-products from yeasts has been reported to improve the attraction of lures for *D. suzukii* [54,55], but the potential for improvement from bacterial fermentation is new and offers an exciting line of inquiry for the future. Bacterially induced fermentation resulted in fine-tuning of certain MVOCs to a level that is highly attractive for SWD. Overall, the chemical studies results show a very complex relationship between bacterial strains and bait features. 

This study, when compared with other studies, found that crucial volatiles for the attractiveness of SWD released as products of malolactic fermentation in the wine-vinegar mixture in the presence of LAB, are commonly found in host fruits as well [14,49,56]. However, some compounds like phenylethyl alcohol, methyl salicylate, and eugenol are also commonly reported as typical plant-green leaf volatiles [14,17]. Moreover, certain compounds have been reported as insect pheromones, e.g., acetoin and 2,3 butanediol [57,58]. Compounds that elicited constant and high electrophysiological response common to all samples belong to classes of acetate esters, esters, acids, short-chain alcohols, and ketones. 

We also found a number of electrophysiologically active compounds for *D. suzukii* (Table 3). Our findings are in line with other electrophysiological studies [14,19,28,49]. Typical yeast volatiles present in DD include ethanol, isobutanol, 3-methyl butanol, 3-hydroxy-2-butanone (acetoin), acetic acid, isobutyric acid, ethyl hexanoate, ethyl octanoate, and phenylethyl alcohol [16,59]. The VOCs of fermenting products and fruit that elicited high and constant electrophysiological responses in our study are: isoamyl acetate, ethyl acetate, isopentyl acetate, hexyl acetate, ethyl hexanoate, benzyl acetate, benzaldehyde, ethyl octanoate, and acetoin [14,60]. Moderate electrophysiological responses resulted from: grape butyrate, 2-phenylethanol, methionol, isoamyl lactate and diethyl succinate, although it is important to remark that none of these volatiles are species-specific. These odors are also well known for eliciting electrophysiological responses in *D. melanogaster* and other drosophilids [14,15,49]. 

An excessive concentration of some compounds characterizing DD could induce positive or negative responses from the olfactory system of the SWD. For example, earlier studies [30,40] reported that several EAD-active compounds released from wine and vinegar had deterrent effects on *D. suzukii* attraction at high concentrations. One of these compounds is isoamyl acetate, likely released by the epiphytic community on fruit surfaces as well as by fermenting substrates [49]. When it was tested, isoamyl acetate was attractive only within a concentration range similar to that emitted by fresh fruit, whereas the 100-fold higher release rates from wine and vinegar were behaviorally repellent [40,49]. In our experiments, the release rate of isoamyl acetate was remarkably reduced after bacterial inoculation and lactic fermentation, which could be one of the reasons for the increased attractivity to *D. suzukii*. It is already established that acetic acid and ethanol are key volatiles for the attraction of *D. suzukii* to wine and vinegar [30].

Our findings further corroborate the commonly accepted theory that the absolute amount of ubiquitous volatiles is the critical factor mediating the recognition and the orientation of polyphagous insects to feeding and oviposition sites [12]. Therefore, the quantitative variations induced in the DD through the addition of LAB should be carefully considered, as well as the interactions of the trap mixture with environmental variables (temperature, humidity, not-target insect catches) during field ageing of lures. 

We show that the emission rate of 3-methyl-1-butanol, reported as a “non-target” attractive volatile for a wide range of moths [53], is higher after addition of strains of *O. oeni* than in the standard DD. However, acetoin, one of the key compounds for the attraction of *D. suzukii* to DD [28,61,62], showed a decrease of about 55% in samples inoculated with LAB. Acetoin is a compound known to be present both in wine and vinegar, and is a fermentation product of LAB [40]. The impact of bacterial activity on the release rate of ester compounds appears more uniform, with a generalized decrease in comparison to DD, except for a slight increase of ethyl octanoate production.

During the first field trial, Trial 1, total catches of *D. suzukii* generally increased from week 1 to week 4. In particular, there was a substantial weekly increase in catch for the positive control, Treatment A. In the treatments inoculated with LAB and left in the field for the entire period of the experiment, liquid bait temperatures appear to have inhibited the development of bacterial flora. Across the four weeks of the trial, Treatments E and F, which contained *O. oeni* Beta, caught more SWD than Treatment D, which contained *O. oeni* Alpha. This suggests that the Beta strain is better adapted to high temperatures than the Alpha strain. Adding citric acid to the mixture in Treatment F resulted in malolactic fermentation leading to increased production of acetoin, diacetyl and 2,3-butanediol [43]. These compounds might increase attractivity to *D. suzukii*. However, traps baited with *O. oeni* and citric acid did not show any change in attraction. The results obtained from Treatment A are most likely due to the weekly replacement of liquid bait making it possible to maintain a higher concentration of autochthonous bacteria. Treatments G and H, which included DD + LAB but had different trap designs, showed very low numbers of captured flies. 

We optimized the trap architecture and bait components in order to keep the temperature of the liquid bait within the optimal range. Different trap designs were tested in the field in order to provide bacteria with optimal growing conditions and allow easier, more rapid release of volatile compounds. Indeed, an innovative trap architecture in combination with the DD + LAB bait produced a powerful attraction and enabled the capture of a greater number of *D. suzukii* compared with other traps, in Trial 3 particularly of females and during the cold periods of low *D. suzukii* population density. This activity could be further improved by calibrating the concentration of *O. oeni* bacterial strains and better controlling the factors that limit the development of these bacteria. We also confirmed that the malolactic fermentation by strains of LAB added to variants of DD appears to be instrumental in boosting catches of *D. suzukii*. It is also clear that, in addition to all other limiting factors such as pH and SO_2_ concentration, a certain temperature must be maintained to ensure that adequate fermentation takes place. For this reason, it is necessary that the mixtures, once prepared, are kept under controlled temperatures and that they are maintained so as not to suffer contamination by other microbial species that could trigger undesirable fermentation. Our results showed that the commercial bacterial strain Enoferm Beta provided the best results, and that the addition of citric acid is a limiting factor for this type of bait. Moreover, a great advantage of the trap developed in this work is that it is capable of being quickly and easily serviced, which helps in planning the use of insecticides and other control strategies in an IPM program approach against SWD.

Contrary to our results, earlier studies suggested that the presence of *O. oeni* cultures did not significantly improve the attraction to DD [33]. However, in these cases, commercial plastic cup traps (Droso Trap^®^) were used, which are highly susceptible to temperature fluctuations that likely inhibited bacterial metabolic activity, as shown in our field trials. Furthermore, the studies were carried out under different environmental and seasonal conditions and used different strains of *O. oeni*.

Monitoring of the target insect is the first step to an IPM program. Ideally, a species-specific trap containing odorants emitted by host fruit during ripening might attract *D. suzukii* earlier in the season, allowing early detection and intervention. Such a trap would help to control the pest on a local level by suppressing population numbers [14,28,63]. The trap could also be utilized in a mass trapping system later in the season. A key goal of mass trapping is to capture the maximum possible number of insects before they reproduce or cause damage to crops. Effective trapping requires the use of lures that are more attractive than natural sources, such as food, female sex pheromones, and mating aggregations. Furthermore, lures should be effective during the entire period of adult emergence and mating [64,65]. Accordingly, the traps must be visually attractive and capable of capturing and retaining flies long enough to provide a lethal dose of toxicant or prevent escape by drowning or starvation [66].

Catching a large proportion of insects during the early season is crucial to reduce the damage caused by *D. suzukii* to the fruit [64,67]. Our results suggest that the use of the *O. oeni*-baited DD trap may provide an important contribution to significantly reduce populations of *D. suzukii*, especially during bottleneck phases under relatively cold climatic conditions of the early growing season. The presence of VOCs in our mixture that are also commonly reported as typical fruit volatiles help to achieve this goal. We hypothesized that population control methods based on behavioral manipulation over a wide territorial scale, such as mass-trapping, attract-and-kill and push-pull, should be maximized near winter shelter areas, as well as in unmanaged environments flanking fruit production areas susceptible to *D. suzukii* infestations [10,68]. In addition, mass trapping methods targeting mainly gravid females and carried out before the beginning of the flowering and fruiting season have the potential to be extremely effective because of the lack of competition between natural sources and bait traps. Further studies are needed to corroborate the results obtained here, in particular under various climatic and environmental conditions, to confirm the possible reduction of *D. suzukii* populations and associated crop damage.

## 5. Conclusions

Overall, our results showed that the attractiveness of wine-vinegar liquid bait for SWD was increased up to two-fold by the addition of commercially available Enoferm Beta strain of *O. oeni* when combined with an innovative trap design. The findings from our studies are currently being utilized for the early detection and mass trapping of *D. suzukii* in Trentino and Alto Adige Valleys, Italy. Future directions will focus on building a better trapping system through utilization of important chemical cues released from *O. oeni* fermentation of wine-vinegar mixtures. The goal is to build a simpler system of attraction in the form of a dispenser with utilization not only of long-range volatiles identified in our study, but also VOCs originating from the leaf canopy of host plants.

## 6. Patents

The use of active culture of lactic acid bacteria for the preparation of a compound for capturing to control of *Drosophila suzukii* and related compounds. Application PCT/IB2015/002054. 

## Figures and Tables

**Table 1:** Trap type and composition of liquid added for three trials, with eight or six treatments per trial. Base solution: DD: Droskidrink + soap; CL: Cha-Landolt solution; pH: KOH added to adjust pH to 3.8. CA—Citric acid (1 g L^−1^). Trap: Cup—Red cup with a white lid; DC—Delta trap with a cup without a lid, with two cotton balls; DIB—Delta trap with an insulated bottle; DUB—Delta trap with the uninsulated bottle; y—Trap replaced weekly; n—not replaced weekly. Codes were created for better separation of treatments in results.

Code	Trial	Base Solution	pH	CA	Alpha/Beta	Alpha/Beta Rate g/L^−1^	Trap	Amount of Liquid (mL)	Liquid Replaced Weekly?
A: Cw	1	DD	2.6				Cup	200	y
A: Cw	2	DD	2.6				Cup	200	y
A: Cn	3	DD	2.6				Cup	200	n
B: Kw	1	DD	3.8				Cup	200	y
B: Kw	2	DD	3.8				Cup	200	y
B: Kn	3	DD	3.8				Cup	200	n
C: ChLn	1	CL	6.5				Cup	200	n
C: ChLw	2	CL	6.5				Cup	200	y
C: ChLn	3	CL	6.5				Cup	200	n
D: α5Kn	1	DD	3.8		Alpha	0.5	Cup	200	n
D: α2Kw	2	DD	3.8		Alpha	0.2	Cup	200	y
D: α2KΔn	3	DD	3.8		Alpha	0.2	DC	15	n
E: β5Kn	1	DD	3.8		Beta	0.5	Cup	200	n
E: β2Kw	2	DD	3.8		Beta	0.2	Cup	200	y
E: β2KΔn	3	DD	3.8		Beta	0.2	DC	15	n
F: β5KnCA	1	DD	3.8	Y	Beta	0.5	Cup	200	n
F: β2KwCA	2	DD	3.8	Y	Beta	0.2	Cup	200	y
F: β2KΔnCA	3	DD	3.8	Y	Beta	0.2	DC	15	n
G: β5KΔIBn	1	DD	3.8		Beta	0.5	DIB	200	n
G: β5KΔCw	2	DD	3.8		Beta	0.2	DC	20	y
H: β2KΔUBn	1	DD	3.8		Beta	0.5	DUB	200	n
H: β2KΔCw	2	DD	3.8		Beta	0.2	DC	15	y

**Table 2:** Relative quantification of volatiles emitted by baits inoculated with different strains of *Oenococcus oeni* (MRI1000, alpha, beta, T1, S18, P1). Data are expressed as the percentage (%) with respect to the amount measured in the standard, unfermented Droskidrink.

Volatile Compound	Retention Time	Extracted Ions	Droskidrink	MRI1000	α	β	T1	S18	P1
	Min	m/z	%						
**Acid**									
Acetic acid	2.2	60	100	47.78	55.75	55.9	874.39	64.88	669.25
**Alcohols**									
Ethanol	1.55	45	100	126.07	129.25	138.499	165.14	119.49	154.68
Isoamyl alcohol	4.7	41	100	123.66	136.69	188.1	115.98	99.73	109.61
**Aldehyde**									
Acetoin	3.78	88	100	149.45	169.16	184.76	78.7	126.11	44.61
**Esters**									
Ethyl acetate	2.27	88	100	5.04	5.53	6.98	3.65	4.33	3.89
2-Butyl acetate	5.55	87	100	114.63	105.54	112.49	70.92	81.63	68.71
Isobutyl acetate	6.17	56	100	70.72	69.19	106.79	48.35	51.06	43.88
Ethyl butyrate	7.36	71	100	64.95	62.2	90.78	52.81	46.44	51.73
Isoamyl acetate	10.84	43	100	40.16	37.22	42.18	31.41	33.42	29.5
Ethyl caproate	16.73	88	100	95.83	92.47	147.36	72.36	57.51	50.62
Ethyl octanoate	25.35	88	100	143.35	135.62	241.42	97.7	91.79	72.75
**Ketone**									
2-Butanone	2.13	72	100	139.39	149.26	164.71	75.32	110.97	84.98

**Table 3:** Compounds that are electrophysiologically active from all eluted mixtures, RT- retention time for compounds eluted on the polar column, retention index from the polar and non-polar column.

Compound Chemical Name		CAS	RT	Retention Index	
			ZB-WAX	ZB-WAX	HP-5 MS
1	Acetic acid	64-19-7	8.32	1480	600
2	2-Butanone, 3-hydroxy	513-86-0	6.02	1288	662
3	3-Methyl-1-butanol	123-51-3	4.9	1117	720
4	Ethyl butyrate	105-54-4	10.58	1591	788
5	Ethyl lactate	97-64-3	6.76	1338	781
6	Butanoic acid	107-92-6	11.15	1630	843.7
7	1-Butanol, 3-methyl-, acetate	123-92-2	4.05	1148	879.8
8	3-(Acetyloxy)-2-butanone	4906-24-5	7.36	1376	899.1
9	1-Hexanol	111-27-3	6.86	1344	862
10	Grape butyrate	5405-41-4	10.11	1518	907
11	Benzaldehyde	100-52-7	9.58	1534	947
12	Ethyl hexanoate	123-66-0	5.26	1234	980
13	Butanoic acid, 3-methyl-	503-74-2	11.57	1668	913.1
14	3-Hexenoic acid, ethyl ester	2396-83-0	6.08	1293	956.8
15	Nonanal	124-19-6	7.6	1392	1074
16	1-Hexanol, 2-ethyl	104-76-7	8.9	1486	1034.9
17	Hexanoic acid	142-62-1	13.98	1833	908.0
18	1- Octanol	111-87-5	10.02	1553	1050
19	Acetic acid, hexyl ester	142-92-7	5.78	1271	1018.1
20	Sorbic Acid	110-44-1	17.74	2133	1089.8
21	Acetic acid, phenylmethyl ester	140-11-4	12.43	1719	1167.3
22	Phenylethyl Alcohol	60-12-8	14.87	1900	1120.1
23	Benzenemethanol, benzyl alcohol	100-51-6	14.4	1865	1039.0
24	Acetic acid, 2-phenylethyl ester	103-45-7	13.63	1793	1262.5
25	Benzeneacetic acid, ethyl ester	101-97-3	13.21	1777	1250.3
26	Eugenol	97-53-0	18.07	2162	1361.5
27	Triacetin	102-76-1	16.85	2059	1354.4
28	Diethyl succinate	123-25-1	11.66	1674	1182.8
29	Benzothiazole	95-16-9	15.62	1960	1225.5
30	Octanoic acid, ethyl ester	106-32-1	8.14	1428	1198.0
31	Benzoic acid, 2-hydroxy-, methyl ester	119-36-8	13.12	1769	1197.8
32	Phenol, 2,4-bis(1,1-dimethylethyl)	96-76-4	19.57	2293	1511.8

## Data Availability

The data presented in this study are available on request from the corresponding author.

## Supplementary Materials

The following are available online at https://www.mdpi.com/2075-4450/12/1/66/s1, Picture S1: Trap types, Figure S1: Trapping experiments with fermentation in open field, Figure S2: Typical GC traces from A) a non-polar column (HP-1) and B) a polar column (ZB-wax), Figure S3: Aligned analyses of the percentages of the chemical compounds, Figure S4: Aligned different CG-MS data chromatographs showing how different strains of lactic acid bacteria changing the volatile profile of the sample, Figure S5: Main chemcial groups in the mixtures, Table S1: Trapping experiments with fermentation in open field.
