# Supplementary material for: Liquid Baits with Oenococcus oeni Increase Captures of Drosophila suzukii
Source: Insects. 2021 Jan 13;12(1):66. doi: 10.3390/insects12010066 (PMC7828427; doi:10.3390/insects12010066)
Supplement: Supplementary file 1 [file insects-12-00066-s001.pdf]

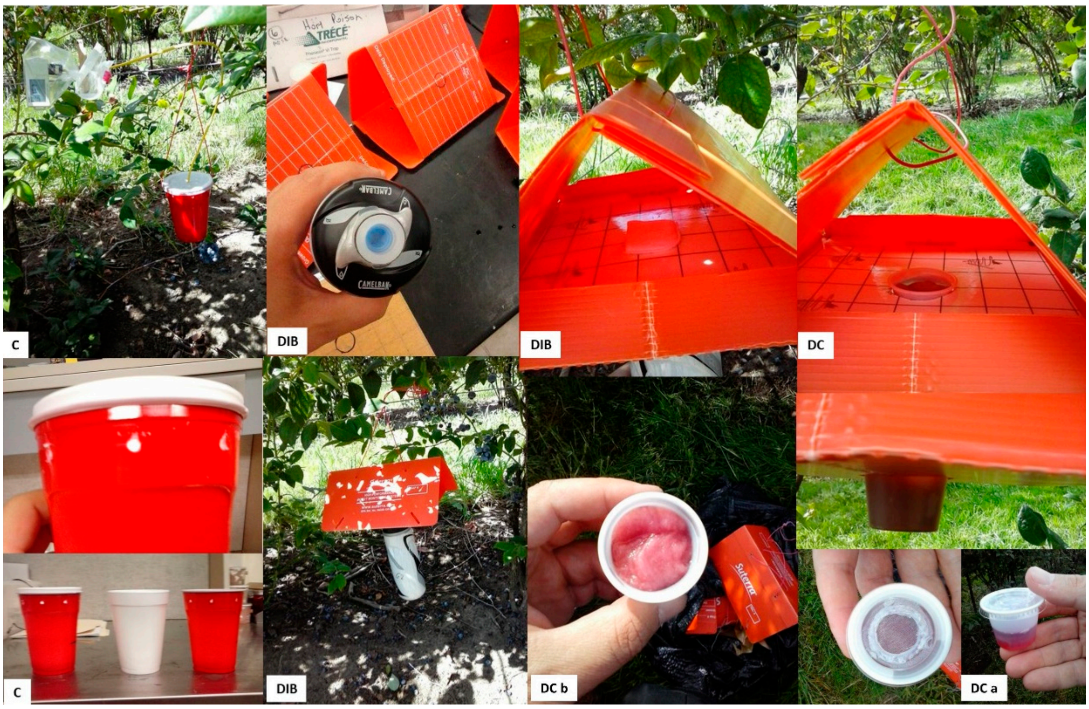

Picture S1. Trap types: C- Red cup with a white lid; DIB- Delta trap with an insulated bottle; DC- Delta trap with a cup without a lid, with two cotton balls.

In the field trials, we wanted to determine which combination of O. oeni strain and trap performed best. In order to create an optimum environment for colonization and reproduction of the bacteria, the pH of the liquid lure was raised to 3.8 using potassium hydroxide (KOH) (monohydrate granular AR [ARS]). Eight treatments were tested in Trial 1.

Trial 1: Treatment A; Droskidrink (DD) natural pH 2.6 in cup shaped trap., B; DD with ajusted pH up to 3.8 in cup shaped trap., C; Che-Landolt solution (Pherocon SWD dispenser, Trécé Inc., Adair, OK, USA) in cup shape trap., D; DD with pH 3.8 with addition of O.oeni strain Alpha in cup shaped trap., E; DD with pH 3.8 with addition of O.oeni strain Beta in cup shaped trap., F; DD with pH 3.8 with addition of O. oeni strain Beta, citric acid in cup shaped trap., G; DD with pH 3.8 with addition of O. oeni strain Beta in DIB trap design., H; DD with pH 3.8, addition of O. oeni strain Beta in delta trap with uninsulated bottle (DUB) trap design. All treatments had 200 ml of liquid tested solution.

Trial 2: In addition, the amount of bacterial inoculum used for the preparation of the treatments was reduced from 0.5 g to 0.2 g L^{-1}. The fermentation of DD by and O. oeni was conducted in the laboratory for one week prior to the field trials under a controlled temperature of 22 ± 2°C. Eight treatments were tested. Treatment A; DD with pH 2.6 in cup shaped trap., B; DD with ajusted pH up to 3.8 in cup shaped trap., C; Che-Landolt solution (Pherocon SWD dispenser, Trécé Inc., Adair, OK, USA) in cup shape trap., D; DD with pH 3.8 with addition of O.oeni strain Alpha in cup shaped trap., E; DD with pH 3.8 with addition of O. oeni strain Beta, citric acid in cup shaped trap., G; DD with pH 3.8, addition of O. oeni strain Beta in delta trap with a cup (DC a) without lead and 20 ml amount of liquid in the cup., H; DD with pH 3.8, addition of O. oeni strain Beta in delta trap with DCb trap design with 15 ml amount of liquid on two cotton balls. Treatments from A to F had 200 ml of tested baits, while treatment G had just 20 ml and treatment H 15 ml amount of liquid.

Trial 3: Trial 3 was the last phase of the fieldwork and involved the assessment and development of a different trap as well as the improvement of traps used in previous trials. This trial used six treatments. Treatment A; DD with natural pH 2.6 in cup shaped trap., B; DD with ajusted pH up to 3.8 in cup shaped trap., C; Che-Landolt solution (Pherocon SWD dispenser, Trécé Inc., Adair, OK,

USA) in cup shape trap., D; DD with pH 3.8 with addition of O.oeni strain Alpha in DC b shaped trap 15 ml of liquid on two cotton balls., E; DD with pH 3.8, addition of O. oeni strain Beta in delta trap with DC b trap design with 15 ml amount of liquid on two cotton balls., F; DD with pH 3.8, addition of O. oeni strain Beta in delta trap with a cup DC b without lead and 15 ml amount of liquid in the cup and citric acid.

Table 1. Trapping experiments with fermentation in open field. Mean counts of *Drosophila suzukii* per day per trap, and % female capture (95% confidence limits).

Trial	Treatment	Male	Female	Total	%Female
	Trial 1				
	A: Cw	39.0 (31.5,48.3)	14.5 (11.6,18.1)	53.5 (43.2,66.2)	27.1 (25.5,28.7)
	B: Kw	23.8 (18.1,31.3)	11.2 (8.7,14.4)	35.0 (26.9,45.5)	32.0 (29.9,34.1)
	C: ChLn	0.2 (0.0,3.8)	0.2 (0.0,1.4)	0.4 (0.0,4.8)	41.5 (22.3,63.6)
	D: Kn	6.6 (3.9,11.1)	3.5 (2.2,5.5)	10.1 (6.2,16.4)	34.5 (30.5,38.6)
	E: 5Kn	16.8 (12.1,23.2)	7.9 (5.8,10.7)	24.6 (18.0,33.7)	32.0 (29.5,34.6)
	F: KnCA	13.0 (9.0,18.8)	6.4 (4.5,8.9)	19.3 (13.6,27.6)	32.9 (30.1,35.9)
	G: 5K IBn	0.7 (0.2,3.5)	0.8 (0.3,2.1)	1.5 (0.4,5.4)	52.0 (41.3,62.6)
	H: K UB n	0.7 (0.1,3.4)	0.6 (0.2,1.8)	1.3 (0.3,5.1)	48.0 (36.6,59.6)
	Trial 2				
	A: Cw	23.1 (15.1,35.3)	14.9 (10.0,22.2)	37.9 (25.2,57.1)	39.2 (35.9,42.5)
	B: Kw	30.0 (20.7,43.6)	18.8 (13.1,26.8)	48.8 (34.0,70.0)	38.4 (35.6,41.4)
	C: ChLw	0.4 (0.0,9.5)	0.2 (0.0,5.8)	0.7 (0.0,14.7)	33.8 (14.7,60.1)
	D: 2Kw	16.7 (10.1,27.4)	11.4 (7.2,18.0)	28.0 (17.4,45.1)	40.6 (36.8,44.5)
	E: 2Kw	32.1 (22.4,45.9)	20.5 (14.6,28.9)	52.6 (37.2,74.5)	39.1 (36.3,41.9)
	F: 2KwCA	22.0 (14.3,34.0)	14.8 (10.0,22.1)	36.9 (24.3,55.8)	40.3 (37.0,43.7)
	G: 5K Cw	6.4 (2.9,14.4)	7.2 (4.0,12.8)	13.6 (6.9,27.0)	52.7 (47.0,58.3)
	H: 2K Cw	9.9 (5.1,18.9)	10.6 (6.6,17.0)	20.4 (11.7,35.7)	51.7 (47.1,56.3)
	Trial 3				
	A: Cn	2.8 (1.8,4.2)	4.2 (2.8,6.3)	6.9 (4.6,10.4)	60.2 (56.1,64.2)
	B: Kn	3.9 (2.7,5.5)	4.4 (3.0,6.6)	8.3 (5.7,12.0)	53.4 (49.7,57.2)
	C: ChLn	0.0 (0.0,*)	0.0 (0.0,*)	0.0 (0.0,*)	100.0 (*,100.0)
	D: 2K n	6.1 (4.6,8.1)	9.6 (7.4,12.6)	15.7 (12.0,20.7)	61.2 (58.5,63.8)
	E: 2K n	10.7 (8.6,13.3)	18.1 (14.9,22.0)	28.8 (23.6,35.2)	62.8 (60.8,64.7)
	F: 2K nCA	7.9 (6.1,10.1)	12.7 (10.0,16.0)	20.5 (16.2,26.0)	61.7 (59.3,64.0)

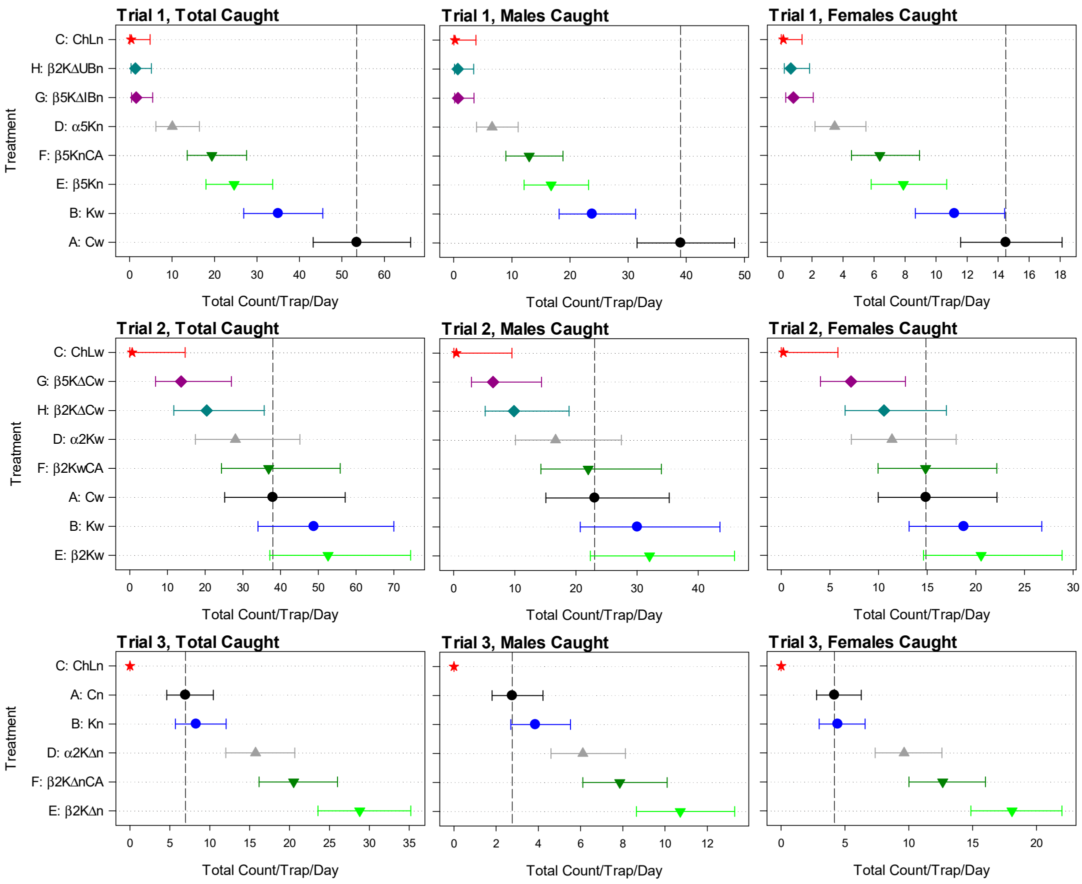

Figure S1. Trapping experiments with fermentation in open field. For each trial, for each treatment: Mean *Drosophila suzukii* catches per trap at each assessment (**a, d, g**); Dot plots of mean total catch trap^{-1} day^{-1} with 95% confidence limits, sorted by the means, with dotted vertical line at the mean for treatment A (control), (**b, e, h**), and percentage of the total catch that was female or male (**c, f, i**). Note that for h, the upper confidence limit for a mean of 0 is not shown as it is difficult to obtain. Treatment codes are as in Table 1: Traps are C cup with a lid;   delta trap; IB: insulated bottle; UB uninsulated bottle. Delta traps without a bottle have a cup without lid with two cotton balls. Liquid components are: K KOH added;   Alpha;   Beta; 2 rate 0.2; 5 rate 0.5; CA citric acid added. n: no Liquid Replacement; w: Liquid replaced weekly.

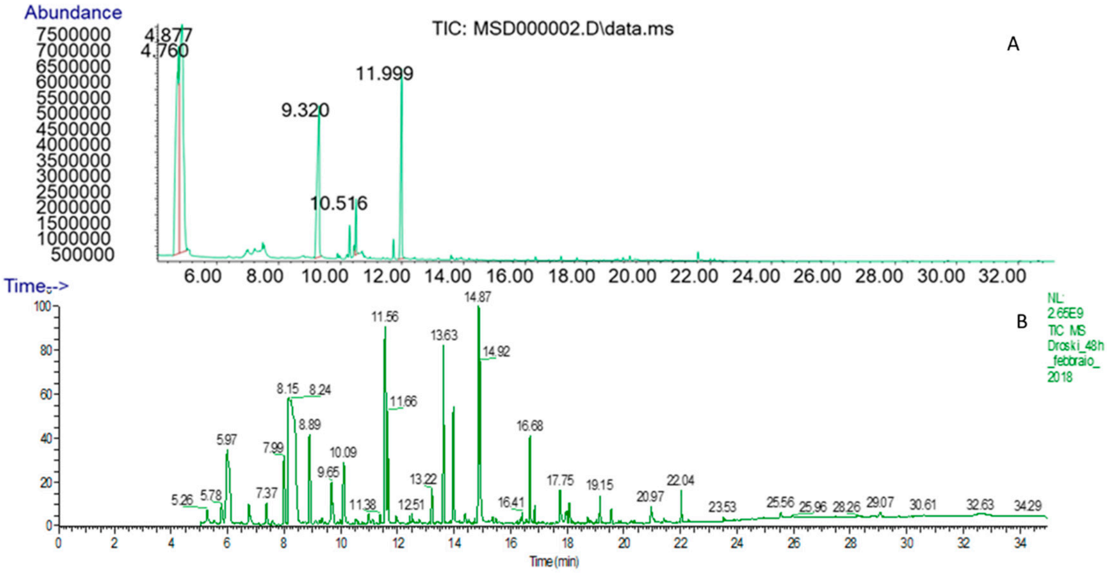

Figure S2. a, b Typical GC traces from (**A**) a non-polar column (HP-1) and (**B**) a polar column (ZB-wax) of 2 µl of an entrainment sample (elution) of headspace volatiles collected from Droskidrink alone.

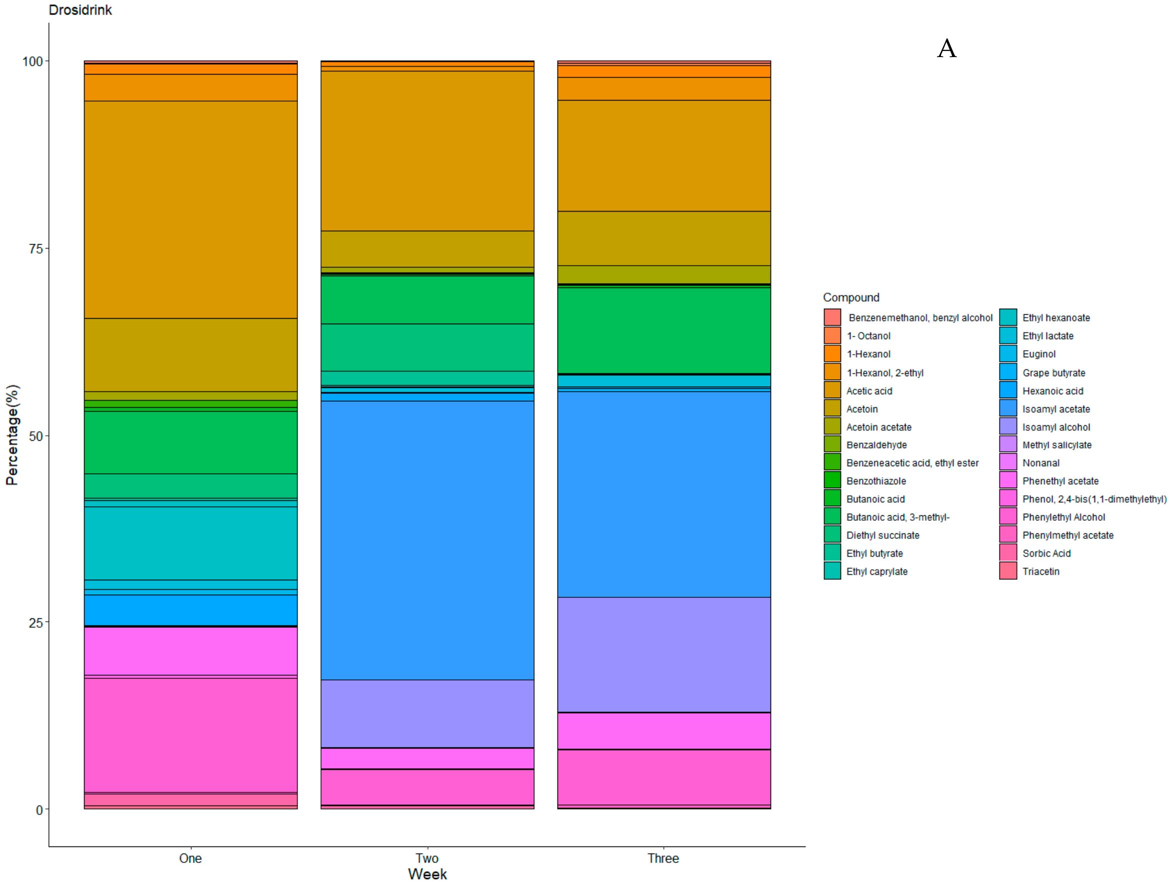

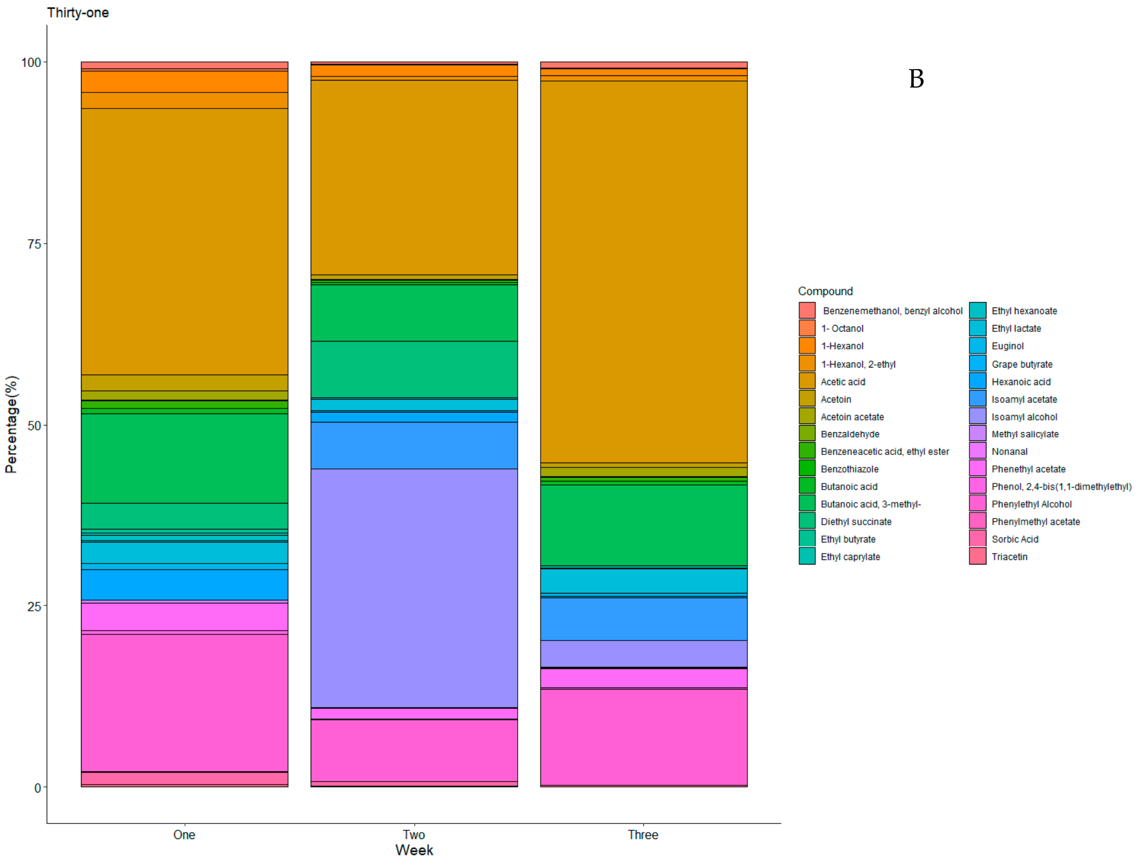

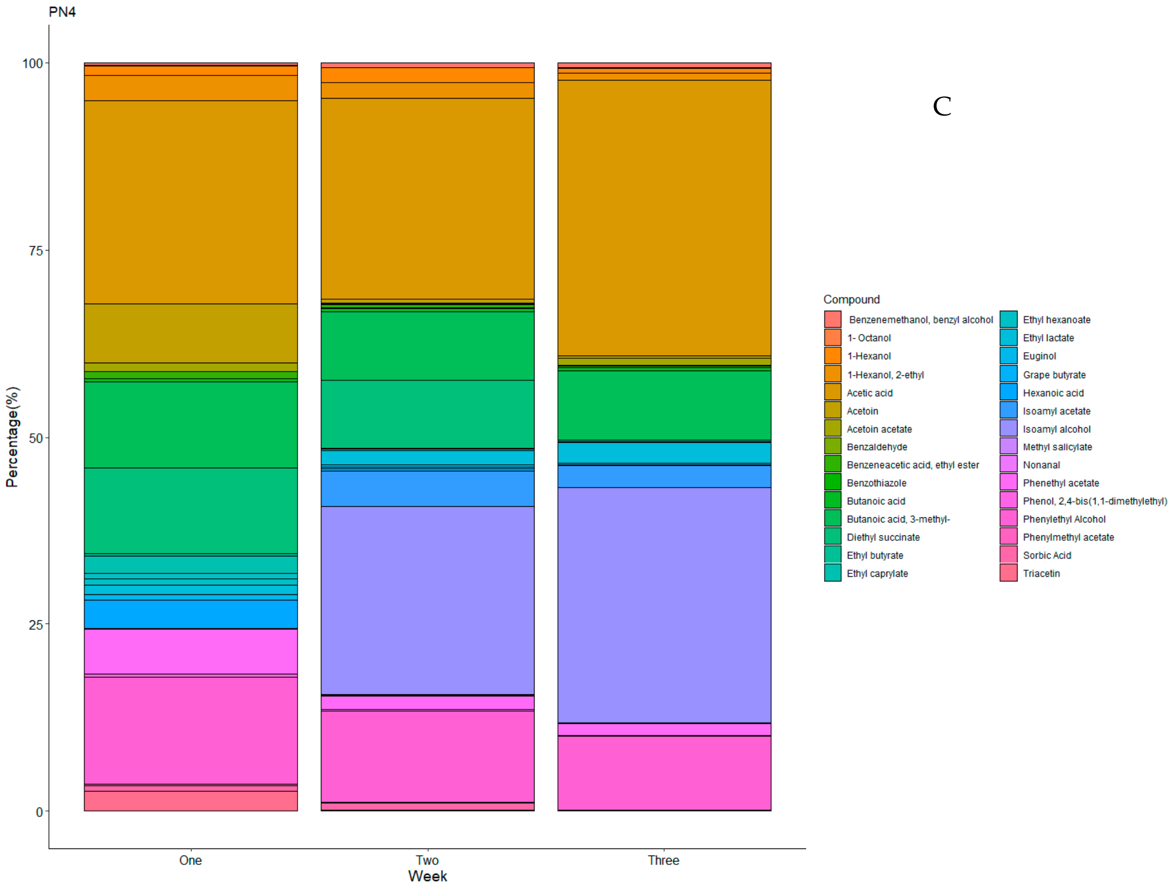

Figure S3. A, B, C. Aligned analyses of the percentages of the chemical compounds. Aligned analyses of the percentages were calculated by dividing the peak area of that compound over the total peak area of the compounds found, a) Droskidrink; b) Oenococcus oeni Strain 31; c) O. oeni strain PN4.

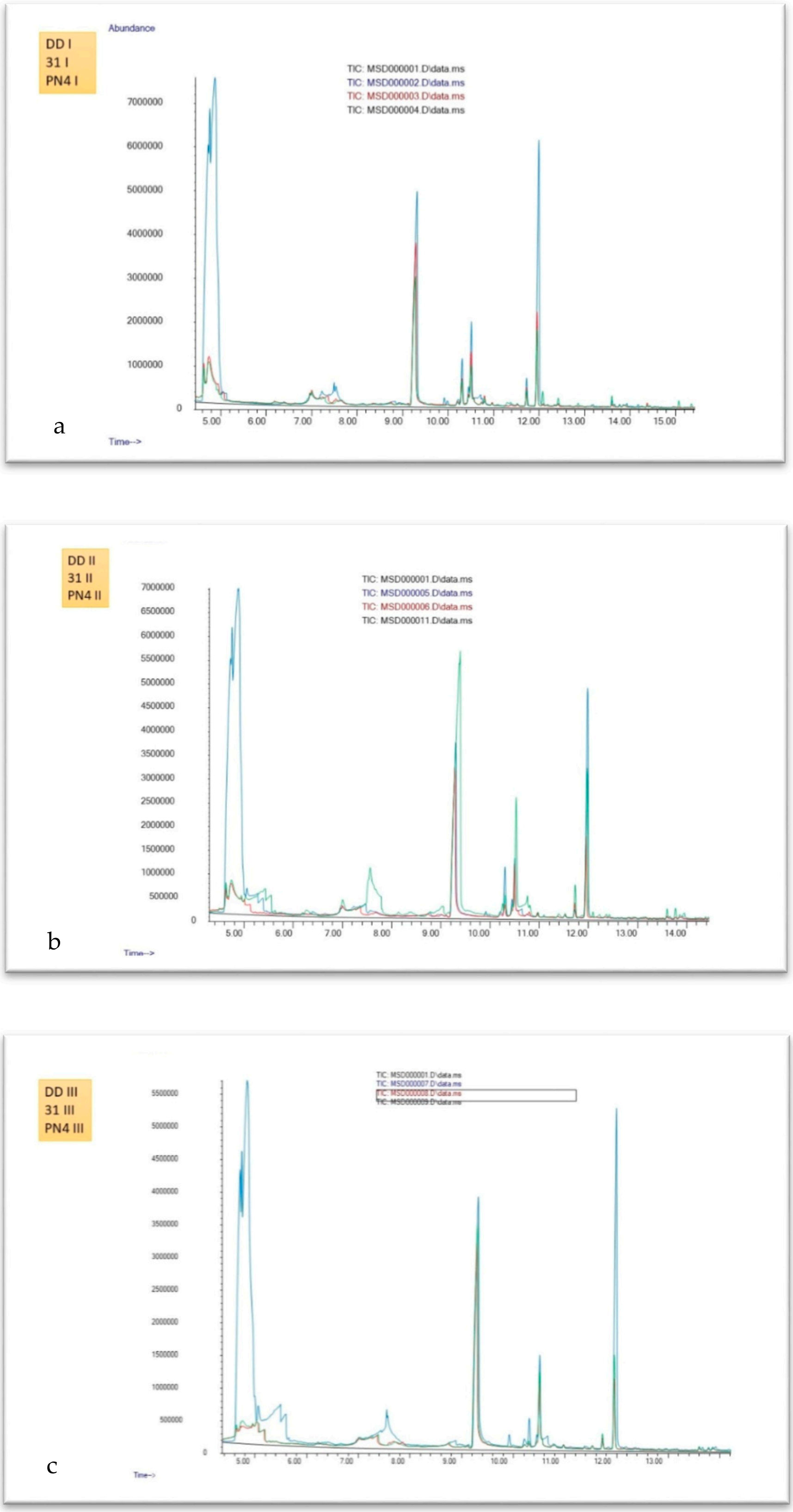

Figure S4. a, b, c. Aligned different CG-MS data chromatographs showing how different strains of lactic acid bacteria changing the volatile profile of the sample.A) 1-week old headspace extracts. DD I: 1 week old Droskidrink; 31 I- with the addition of Oenococcus oeni strain 31; PN4 I- droskidrink with the addition of O. oeni strain PN4. B) 2-week old headspace extracts. DD II: 2 week old DD; 31 II: DD with the addition of O. oeni strain 31 2 week old; PN4 II: DD with the addition of O. oeni strain

PN4 2 week old. C) 3-week old headspace extracts. DD III: 3-week old DD; 31 III: DD with the addition of O. oeni strain 31 3-week old, PN4 III: DD with the addition of O. oeni strain PN4 3-week old.

A:

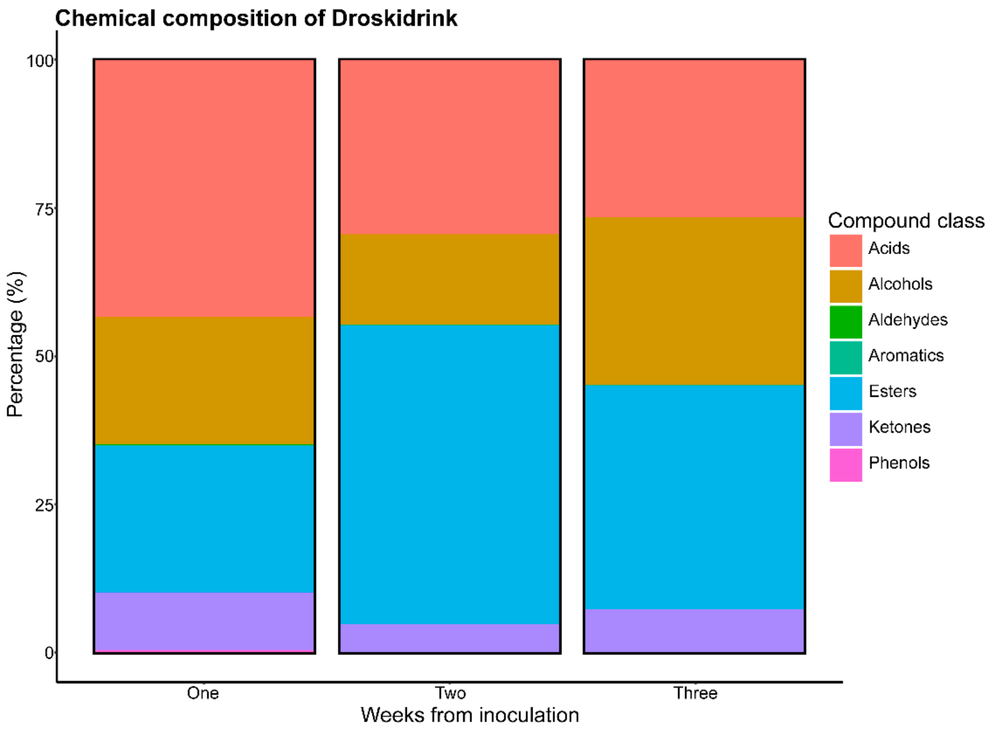

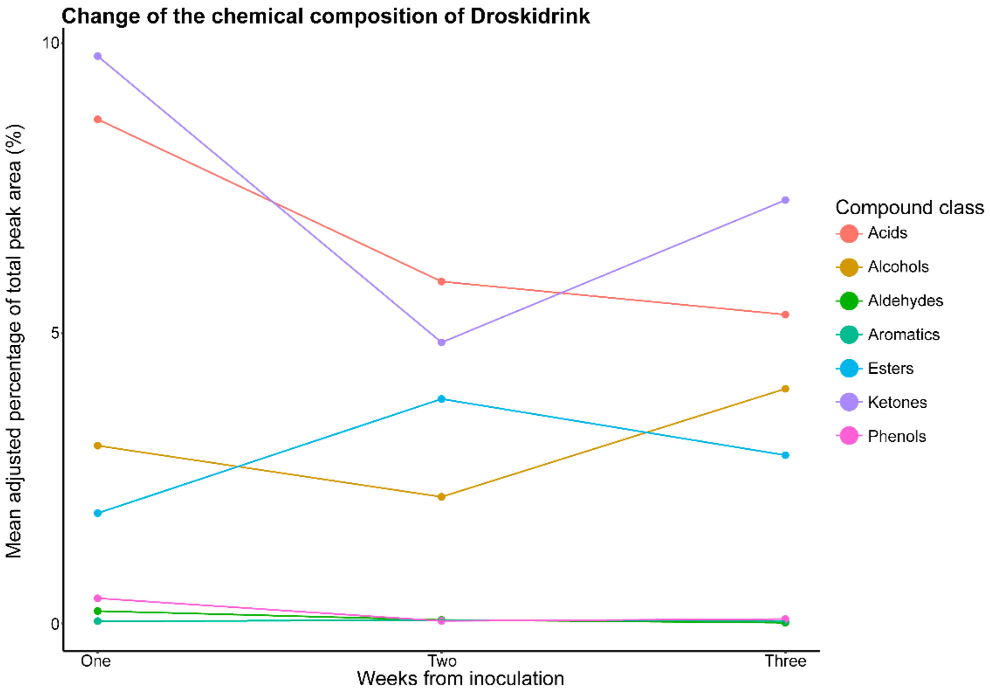

B:

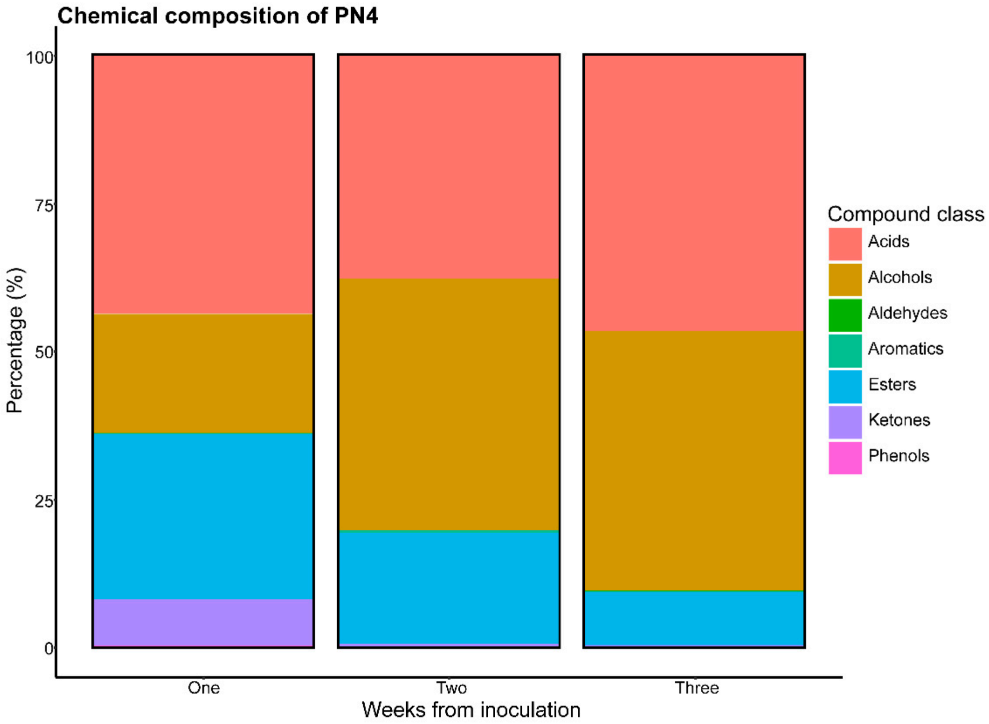
Chemical composition of PN4
Percentage (%)
100
75
50
25
0
One
Two
Three
Weeks from inoculation
Compound class
Acids
Alcohols
Aldehydes
Aromatics
Esters
Ketones
Phenols

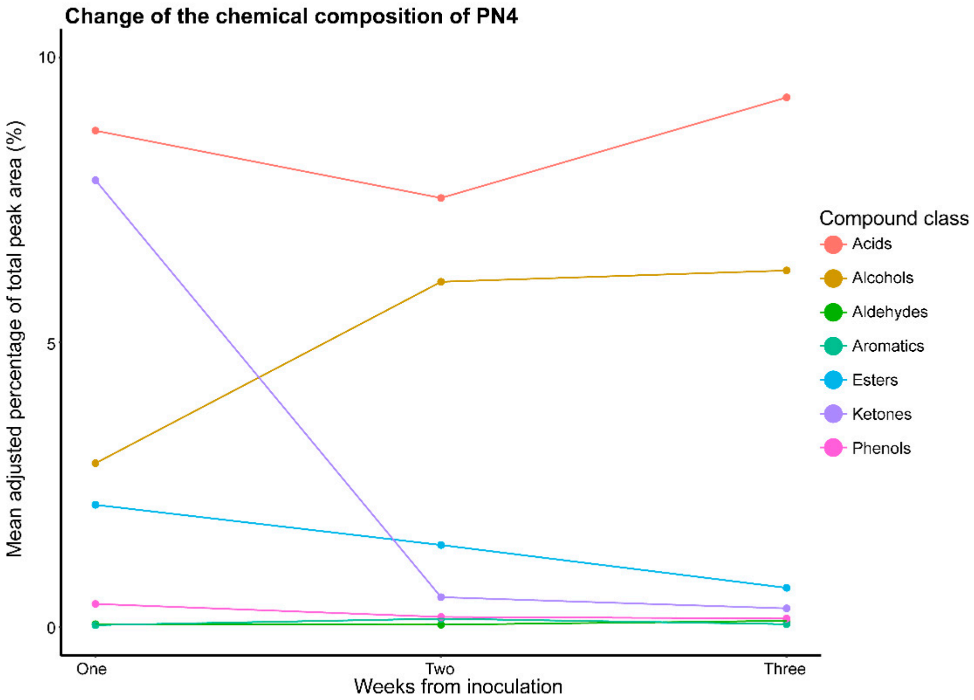
Change of the chemical composition of PN4
Mean adjusted percentage of total peak area (%)
10
5
0
One
Two
Three
Weeks from inoculation
Compound class
Acids
Alcohols
Aldehydes
Aromatics
Esters
Ketones
Phenols

C:

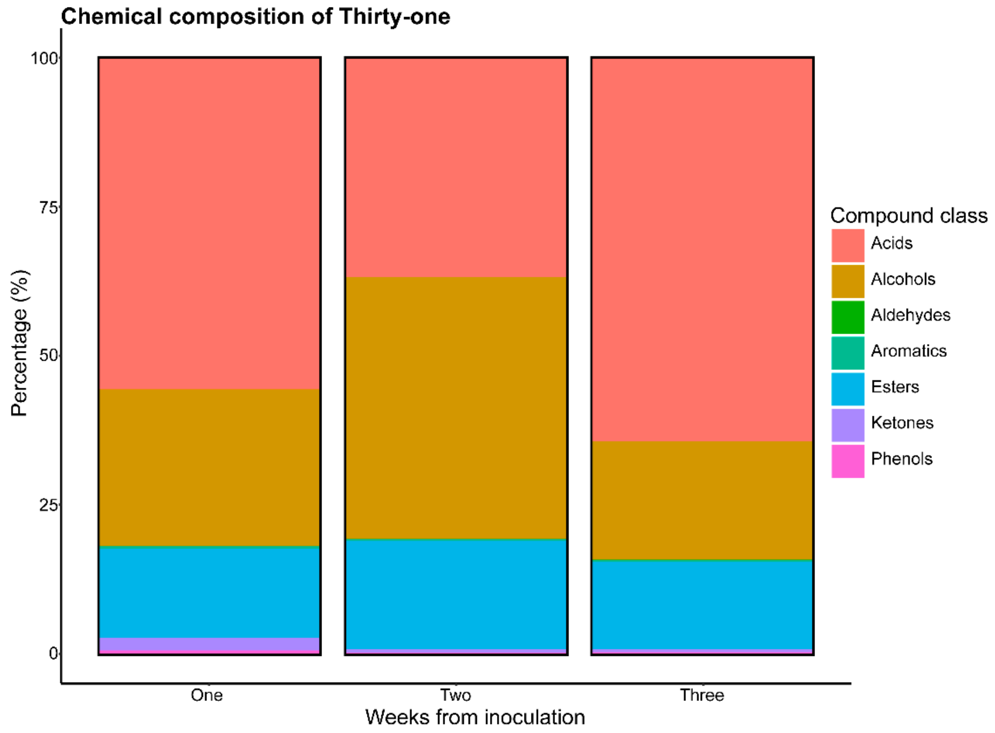

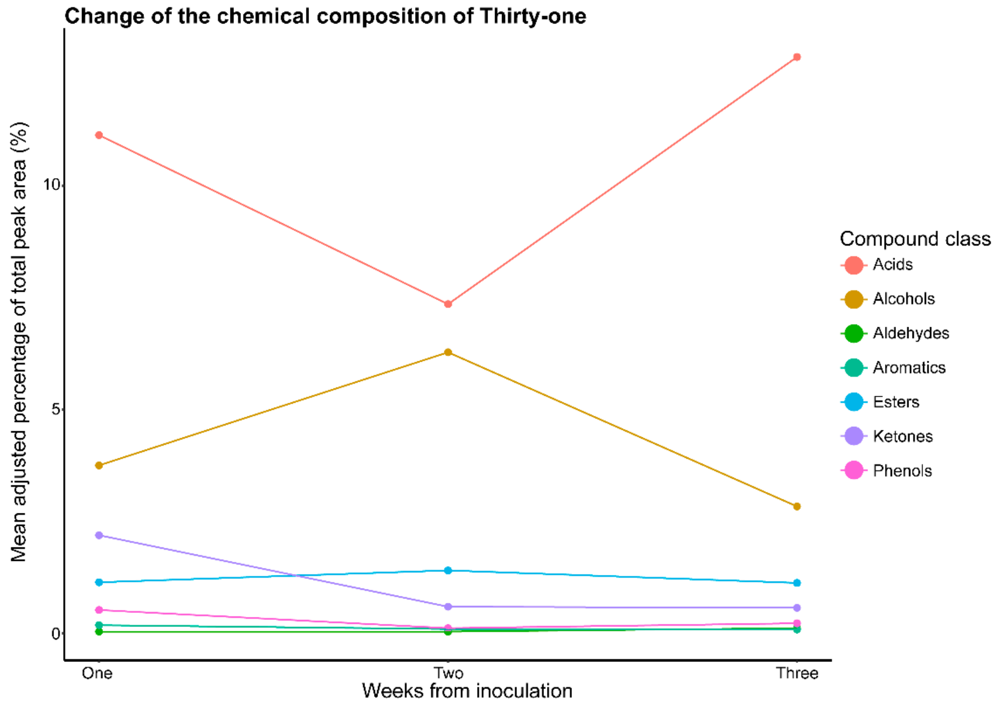

Figure S5. A, B, C. Main chemcial groups in the mixtures. Comparison with main chemical groups present in Droskidrink **a** and their change under influence of Oenococcus oeni and fermentation time. Change of chemical composition in droskidrink with the addition of strain PN4 of O. oeni **b**; change of chemical composition in Droskidrink with the addition of strain 31 of O. oeni **c.** Compound belonging to the same chemical group and their total peak area were compared between samples.
